# OMG: Open Molecule Generator

**DOI:** 10.1186/1758-2946-4-21

**Published:** 2012-09-17

**Authors:** Julio E Peironcely, Miguel Rojas-Chertó, Davide Fichera, Theo Reijmers, Leon Coulier, Jean-Loup Faulon, Thomas Hankemeier

**Affiliations:** 1 TNO Research Group Quality & Safety, P.O. Box 360, NL-3700, AJ, Zeist, The Netherlands; 2 Leiden/Amsterdam Center for Drug Research, Leiden University, Einsteinweg 55, 2333, CC, Leiden, The Netherlands; 3 Netherlands Metabolomics Centre, Einsteinweg 55, 2333, CC, Leiden, The Netherlands; 4 iSSB, Institute of Systems and Synthetic Biology, University of Evry, Genopole Campus 1, Genavenir 6, 5 rue Henri Desbruères, EVRY, 91030, Cedex, France

**Keywords:** Computer assisted structure elucidation, Structure generator, Molecule generator, Open source, Chemical structures, Metabolite identification, Metabolite, Metabolomics

## Abstract

Computer Assisted Structure Elucidation has been used for decades to discover the chemical structure of unknown compounds. In this work we introduce the first open source structure generator, Open Molecule Generator (OMG), which for a given elemental composition produces all non-isomorphic chemical structures that match that elemental composition. Furthermore, this structure generator can accept as additional input one or multiple non-overlapping prescribed substructures to drastically reduce the number of possible chemical structures. Being open source allows for customization and future extension of its functionality. OMG relies on a modified version of the Canonical Augmentation Path, which grows intermediate chemical structures by adding bonds and checks that at each step only unique molecules are produced. In order to benchmark the tool, we generated chemical structures for the elemental formulas and substructures of different metabolites and compared the results with a commercially available structure generator. The results obtained, i.e. the number of molecules generated, were identical for elemental compositions having only C, O and H. For elemental compositions containing C, O, H, N, P and S, OMG produces all the chemically valid molecules while the other generator produces more, yet chemically impossible, molecules. The chemical completeness of the OMG results comes at the expense of being slower than the commercial generator. In addition to being open source, OMG clearly showed the added value of constraining the solution space by using multiple prescribed substructures as input. We expect this structure generator to be useful in many fields, but to be especially of great importance for metabolomics, where identifying unknown metabolites is still a major bottleneck.

## Background

Computer Assisted Structure Elucidation (CASE) of chemical compounds is one of the classical problems positioned at the intersection of informatics, chemistry, and mathematics. CASE tools have been employed during decades to elucidate the chemical structure of small organic molecules. In its most general definition, a structure elucidation system receives experimental chemistry data of an unknown molecule as input, and outputs a list of possible chemical structures. The input can be the elemental composition of the elusive molecule, nuclear magnetic resonance (NMR) and/or mass spectrometry (MS) spectra (provided the generator can simulate spectra and match it to the experimental ones) or information of prescribed substructures. The output is a list of candidate structures matching these conditions, ideally containing all possible structures without duplications. A small list of candidates is dependent on the number of constraints derived from experimental data; the higher the number of constraints we use the smaller the candidate list will be. The ultimate goal for such a system being fully automated and returning only one and correct molecule is not yet at our reach, despite decades of research [1].

The DENDRAL [2] project is widely regarded as the initiator of the use of these methods to provide a system for Computer Assisted Structure Elucidation (CASE). It involved the development of artificial intelligence algorithms that would extract heuristics from MS and NMR data and use them to constrain the output of a structure generator. CONGEN was the structure generator developed within DENDRAL, which preceded a more advanced generator known as GENOA [3]. Many commercial structure generators were developed later, most renowned ones being CHEMICS [4], ASSEMBLE [5], SMOG [6], and the most widely used of all of them, the general purpose structure generator MOLGEN [7]. These closed source software tools work like a black box, where the user cannot, on the one hand, understand the functioning of the software and on the other hand, customize the tool to his needs. These drawbacks of closed source software (where the source code is not provided) can be circumvented by open source tools. Two open source structure generators have been developed that work with NMR data, the deterministic LSD [8] and the stochastic SENECA [9]. Implementation of open source stochastic and deterministic structure generators have been explored within the Chemistry Development Kit (CDK) [10,11]. Unfortunately, these generators failed to generate all chemical structures possible and were discontinued in recent releases of CDK. Despite these efforts, no general purpose deterministic structure generator has been developed in an open source format so far.

The advance of “omics” sciences in the last decade, in particular of metabolomics [12], has renewed the interest of researchers in developing better structure generators. Metabolomics aims at detecting and identifying metabolites in an organism and has resulted in a large list of potential biomarkers for which the chemical structure is unknown [1,13]. When trying to identify the structure of unknown molecules, scientists first perform an identity search by querying reference databases using their experimental information [1,14-16]. In such case, they use the elemental composition of the metabolite derived from mass spectrometry (MS) or the spectra of nuclear magnetic resonance (NMR). When the metabolite is a real unknown it is not present in any database, therefore the query returns no results. This forces scientists to propose candidate structures using a different approach, one of them is using a structure generator [17,18], which produces all possible molecules given an elemental composition and optional, other constraints. Examples of constraints are prescribed substructures that each output molecules should contain and that are derived from experimental NMR, MS^2^, or MS^n^ data. Hence, the need for deterministic and flexible structure generators in the field of metabolomics presents should be met with new algorithms [1].

The majority of structure generators rely on graph theory to produce their desired output. Interestingly, compounds can be represented as molecular graphs where atoms and bonds are translated into vertices and edges, respectively, to which theorems and algorithms proposed by graph theory can be applied. This ensures that the output is correct, exhaustive, and free of isomorphs. Such methods can be the orderly enumeration proposed by Read [19] and Faradzev [20], a stochastic generator [21], the homomorphism principle [22] used by MOLGEN, or the “canonical augmentation path” proposed by McKay [23]. This last method, originally intended to generate simple graphs by adding vertices, has been applied to the generation of some families of graphs and also to generate the chemical universe of molecules up to 11 atoms [24] and recently to 13 atoms [25]. Despite the goal was to generate molecules, these two approaches initially employed canonical path augmentation to generate all possible simple graphs up to 11 and 13 vertices, respectively. Posterior topological and ring system filter were used to remove unwanted graphs. Lastly, the vertices were colored with chemical elements and the edges with a bond order, which turned the graphs into molecules. Simple chemical constraints like connectivity and atom valence were applied to reduce the list of final molecules. This process, which relies on generating simple graph, is necessarily limited on the size of the molecules that can be generated because a linear increase in the number of atoms produces an exponential increase of both the number of graphs and molecules. Here we present the Open Molecule Generator (OMG), a structure generator based too on McKay augmentation algorithms, but rather than first generating graphs and secondly transforming these graphs into molecules, our implementation of McKay technique directly constructs molecules. In this way we can generate chemical structures much greater than 13 atoms. Essential concepts of graph theory will be introduced in the methods section.

Chen mentioned two future challenges facing CASE systems [26]. The first challenge for elucidating structures is to have a knowledge system of previously identified compounds, as well as mining tools for such data. In this direction, Rojas-Chertó et al. [27] developed a system to store spectral data and mine the database to extract substructure information that can be used as prescribed substructures in our structure generator. The second challenge is the need for filtering and selecting candidate structures. This is often performed by predicting a property of the candidate structures that is related to the field of research, for instance, predicting the spectra in analytical chemistry, the bioactivity in ligand design, or the Metabolite-Likeness [28] in metabolomics studies, to name a few. Furthermore, the need of a structure generator tool that can be adapted to the requirements of the field in which it is going to be applied, demonstrates the usefulness of open source tools compared to commercial "black box" generators.

In this paper we present the first general purpose open source structure generator, Open Molecule Generator. OMG adapts methodologies from the field of graph theory and deterministic graph enumeration to the classical problem of chemical structure generation. In this sense, we have used the approach of “canonical path augmentation” to ensure that we exhaustively generate non-isomorphic chemical structures for a given elemental composition. This generation tool has been implemented using CDK [10,11], a widely used open source library for the development of chemoinformatics software. It allowed the representation of entities such as molecules, atoms, and bonds in our program and the use of functions like removing hydrogen atoms, checking the saturation of a molecule, removing a bond, and many more. The resulting tool generates all possible non-duplicate chemical structures for a given elemental composition, with the option to generate only those that contain one or multiple non-overlapping substructures, which is the most important constrain to reduce the number of resulting candidate structures when a knowledge system is not available [18]. We have used OMG to generate molecules for the elemental composition of well known metabolites, also including one or more prescribed substructures as input. These results are compared to those obtained by MOLGEN.

## Materials and methods

### Chemical elements and atom types

We would like to describe some concepts related to atoms that are necessary to understand the theory and algorithm behind OMG and the use of CDK to handle chemistry.

In nature, atoms of different chemical elements (carbon, nitrogen, oxygen, and others) are connected to each other by bonds in order to form molecules. The valence, to which we will also refer as degree, of these chemical elements determine how many bonds each element can have. Carbon has a valence of 4, oxygen of 2, nitrogen of 3 or 5, sulfur of 2,4 or 6, phosphor of 3 or 5. Thus a carbon atom becomes saturated when it has 4 bonds, where a single bond counts as one bond, a double as two bonds, and a triple as three bonds. Regarding molecules, we consider a molecule to be saturated when all its atoms are saturated. In some special occasions, atoms are charged, which makes them having a different valence. In the case of OMG, we only use neutral atoms and as a consequence only neutral molecules are produced, therefore all finished molecules will contain atoms with the valences mentioned before.

A chemical element can have multiple atom types, also for the same valence of an element, as defined by the dictionary of atom types in CDK. This dictionary defines for each atom the number of neighbors, pi bonds, charges, lone electron pairs, and hybridizations, in order to accommodate the different states a chemical element can have due to different bonds, number of neighboring atoms, charges and hybridizations. These atom types are based on the chemical elements that have been observed in nature for saturated molecules. This is why we use the CDK atom dictionary to validate the atoms of our finished molecules.

OMG will output only molecules that are saturated and that contain the atoms specified in the elemental composition. Apart from finished molecules, OMG has to represent during the generation process intermediate chemical structures that are not finished yet. These might contain disconnected fragments and atoms that are not saturated. CDK atom types are not designed to represent atom types of unsaturated chemical elements; therefore we opted for implementing a simple atom dictionary. For each chemical element, this dictionary defines its valence, in other words, the maximum degree. Hence for intermediate chemical structures we only check that the current degree of each atom does not exceed the maximum degree.

MOLGEN can also produce molecules with multiple valences, but it handles them in a different way. While with OMG only the elemental composition needs to be provided to generate molecules with multiple valences, MOLGEN requires knowing a priori which one of the multiple valences has to be used. It uses by default the lowest valence, this is, N valence 3, P valence 3, and S valence 2, unless a different valence is specified. In Table 1 the atom types produced by OMG and MOLGEN for non-default valences are presented. Using sulfur as an example, OMG will output molecules with containing sulfur valence 2, 4 and 6. For the same chemical element, MOLGEN will produce by default molecules with sulfur valence 2. If one sets the valence of sulfur to 6, it will only produce sulfur valence 6 and not valence 2 and valence 4. MOLGEN cannot generate molecules with atoms of different valences for the same chemical element, this is, if molecule has two sulfur atoms, one will not be of valence 4 and the other of valence 6, both will be either valence 2, 4 or 6.

**Table 1:** Atom types produced by OMG and MOLGEN for non-default valences of N(5), P(5) and S(4 and 6)

**Valence**	**MOLGEN**	**OMG**
**N valence 5**		
**P valence 5**		
**S valencee 4**		
**S valence 6**		

By default OMG outputs molecules with valences N(3 and 5), P(3 and 5), and S (2,4 and 6). By default MOLGEN outputs molecules with valences N(3), P(3), and S(2).

The principle followed by CDK to build its atom dictionary is to allow atom types with valences for which there is a consensus agreement on their existence, this is, for which known molecules exist with such valences. Conversely, MOLGEN produces all theoretically possible combinations of bond orders for a given valence, as it can be observed in Table 1. For example, as it can be seen for P valence 5 OMG only produces one atom type with one double bond and three single bonds. In comparison, MOLGEN produces all the combinations of single, double, and triple bonds that add to 5. As a consequence, when the desired valence is unknown, which is usually the case in metabolite identification, molecules need to be generated with all possible valences. As a result, the number of output molecules by both generators is different for elemental compositions that contain chemical elements with multiple valences. This deterministic generation of valences in MOLGEN comes at the expense of generating molecules having unrealistic structures.

### Graph theory and chemistry

The chemical structure of molecules can be represented as a graph, where atoms and bonds in molecules correspond to vertices and edges, respectively, in graphs. In molecules, bonds connecting two atoms can have a degree depending on the number of electrons they share. Such a degree can also be assigned to the edges of a graph, which is called a multigraph. The different chemical elements present in the periodic table are represented in graphs as colors assigned to the vertices. We define a non-directed colored multigraph *G* = (*V*, *E*) as where *V* is a set of vertices and *E* is a multiset of edges, where each edge is an unordered pair of vertices, and a function *Col* : *V* → *colors*. In this multigraph, we say that *a*, *b* ∈ *V* are *n-connected* if there are exactly *n* edges (*a*, *b*) ∈ *V*. Apart from the color function, a multigraph is characterized by the function *d* : *V* × *V* → *N* which returns the degree of the edge connecting each couple of vertices. From now on we will indistinctively refer to graphs and multigraphs.

In chemistry, the valence rule determines the maximum number of bonds each chemical element has. In order to take this into account, we define *d*_*v*_ : *V* → *N* which returns the number of edges of a given vertex and a *max-degree* function *md* : *V* → *M*, which returns the maximum number of edges of a given vertex. We say that a multigraph is under-saturated if ∀ *v* ∈ *V*, *d*_*v*_(*v*) ≤ *md*(*v*) there is at least one vertex such that *d*_*v*_(*v*^′^) < *md*(*v*^′^). A multigraph is saturated if the equality *dv* = *md* holds for every vertex. In chemistry, molecules correspond to saturated colored multigraphs and *max-degree* depends on the color, which is the chemical element. For instance, for a carbon element, *md*(*C*) = 4  and for an oxygen element, *md*(*O*) = 2.

We consider a multigraph to be connected if ∀ *v*, *w* ∈ *V*,  ∃ *S*_{*V*,*W*}_ = {*v*_1_, ⋯, *v*_*m*_} such that *v*, *v*_1_ and *v*_*m*_, *w* are connected and for each *i* < *m*, *v*_*i*_ is connected to *v*_*i* + 1_. In other words, a multigraph is connected if for all pair of vertices, there exists at least one path *S*_{*V*,*W*}_ connecting both vertices. This condition is necessary for chemistry, since intermediate chemical structures in the generation process can be composed of disconnected fragments, it ensures that the generated molecules are one fully connected structure and not made of disconnected substructures. Notice that hydrogen atoms (the most frequently found chemical elements with degree 1) are not considered in the generation process, since they are terminal elements of the molecule and they cannot connect two disconnected elements of the molecule. Hydrogen atoms are only used to validate the completeness of finished molecules. Halogen atoms like fluorine, chlorine, and iodine, also of degree 1, are considered during the generation process.

### Graph labeling

An isomorphism π is a function that for each vertex *v* ∈ *V*, *Col*(*π*(*v*)) = *Col*(*v*) and for each pair of vertices *v* ∈ *V*, *v* ^′^ ∈ *V* ^′^, *d*(*π*(*v*), *π*(*v*^′^)) = *d*(*v*, *v*^′^). A labeling function *σ* : *V* → {1, ⋯, *n*} is a bijective map from the vertices of a colored multigraph to an ordered list labels with a cardinality equal to the number of vertices. Put simple σ, assigns to each vertex a label. Let *σ*^− 1^ be the inverse function of σ, which returns the vertex corresponding to a label. We say a labeling function is canonical if given any two isomorphic colored multigraphs *G* = (*V*, *E*) and *G* ^′^ = (*V* ^′^, *E* ^′^), the bijective function *π* : *V* → *V* ^′^ defined as *π*(*a*) = *σ*^− 1^(*σ*(*a*)) is an isomorphism of *V* in  *V* ^′^. Therefore, a canonically labeled multigraph is a multigraph whose vertices are associated to an ordered list through a canonical labeling function. Furthermore, a canonical hash of the labeling is a bijective function between the space of the canonically labeled multigraphs and the value space and it is represented as a string of integers. It is interesting to note here that two isomorphic graphs have the same canonical hash, a fact that will be used to remove duplicated molecules during the generation process.

### Using fragments

A fragment or substructure of a molecule is equivalent to a fragment or subgraph of a graph. We define a fragment as a subset of a graph and it is characterized by the function *d*_*f*_ : *V* × *V* → *N* where *N* is the number of edges connecting each pair of vertices in the subgraph. Such *d*_*f*_ has to fulfill the condition *d*_*f*_(*a*, *b*) ≤ *d*(*a*, *b*), ∀ *a*, *b* ∈ *V* and at least for one edge *d*_*f*_ < *d*, this is, the fragment should have fewer edges than the graph.

### Canonical augmentation

An augmentation of a multigraph *G* = (*V*, *E*) is a multigraph *G*^′^ = (*V*^′^, *E*^′^), defined on the same set of vertices, such that $\forall a,b\in V,d_{G}\left(a,b\right)=d_{G^{\prime }}\left(a,b\right)$, except for one and only one pair where  *d*_*G* ′_(*a*, *b*) = *d*_*G*_(*a*, *b*) + 1. Let *e* ^′^ ∈ *E* ^′^ be the edge which degree has been increased, *d*(*e*^′^) = *d*(*e*) + 1.

Let *e*^*c* ′^ be the last edge of *σ*(*G*^′^) and *a*^*c* ′^, *b*^*c* ′^ ∈ *V* the vertices of *e*^*c* ′^. Consider *σ*^− 1^(*a*^*c* ′^, *b*^*c* ′^) = *a* ^′^ ^′^, *b* ^′^ ^′^ to vertices of *G* ^′^ ^′^, a copy of *G*^′^, to which a bond order decrease is performed *d*(*a*^′ ′^, *b*^′ ′^) = *d*(*a*^′ ′^, *b*^′ ′^) − 1. The resulting multigraph *G*^′ ′^ after this decrease in bond order, can be seen as the result of a canonical deletion on *G*^′^, the reverse operation of a canonical augmentation. In our definition of canonical augmentation we consider a multigraph *G*^′^ = (*V*^′^, *E*^′^) to be canonically augmented from *G* = (*V*, *E*) if it is an augmentation and *σ*(*G*^′^ ^′^) = *σ*(*G*). In other words, we consider *G*^′^ to be a canonical augmentation of *G* if a canonical deletion in *G*^′^ results in *G*.

### Description of the algorithm

The generation of structures can be seen as a tree of intermediate chemical structures that our tool explores. At the root of the tree we find a collection of fully isolated/disconnected atoms. One bond is added at each level of the tree, resulting in fully connected/finished molecules at the leaves. The canonical augmentation path is a depth-first backtracking algorithm, where the recursive function *generate* described in Algorithm 1, implements the addition of one bond in all possible ways for a given intermediate chemical structure, and evaluates for each extended molecule that this extension has been performed in a canonical way, as described before. Here adding one bond means increasing the degree of the bond between two atoms, hence a single bond becomes a double bond and a double bond becomes a triple bond. If there is no bond between two atoms, a single bond is created.

Between lines 2 and 9 of Algorithm 1, the molecule is stored if it is finished, which occurs when the molecule is saturated and all the atoms of the elemental composition, including the hydrogen atoms, have been used, all the atoms are validated by the CDK atom dictionary and are connected forming one single structure and not multiple disconnected fragments.

In the case the molecule is not finished, it would be extended in all possible ways by adding one bond. If there exists a bond between a pair of atoms function *extend*, in line 12 of Algorithm 1, will increase the multiplicity. The generation of new bonds is controlled by OMG atom type definitions for intermediate chemical structures, which guarantee that the degree of the atoms does not exceed the maximum degree allowed for its chemical element.

Function *canonize*, in line 15 of Algorithm 1, returns the canonical version of the molecule. We modified the graph canonizer Nauty [23,29] in order to allow multigraphs and not only simple graphs. Other canonizers for graphs exist like MOLGEN-CID [30] or the Signature Canonizer [31], but Nauty has been the most widely used for graphs as well as for chemistry problems, like InChI [32] codes. Nauty is the canonizer of choice because it is the fastest of all available canonizers for bounded valence graphs below 100 vertices [33] (molecules are examples of this class of graphs). Firstly, the function *canonize* translates the molecule into a colored multigraph. Secondly, it utilizes Nauty to calculate the canonical labeling of the multigraph. Thirdly, this canonical labeling is used to construct the canonical version of the input molecule. Lastly, the canonical hash string of each augmented molecule is stored in a hash map, lines 16 and 17, in order to remove duplicated extensions at each level of the tree. Each unique extension is checked for canonical augmentation, line 18, using Algorithm 2, or Algorithm 3 in case prescribed substructures were provided. If this extension is successful, the function *generate* is called, line 19 of Algorithm 1, and the molecule we want to continue extending is passed as a parameter. When a molecule cannot be extended any further, the recursion is terminated and the program backtracks in the search tree.

### Input and output

The minimum input required is the elemental composition of the structures that have to be generated. Optionally, a structure-data file (SDF) can be provided containing one or more prescribed substructures that we want our output molecules to contain. Since OMG does not take hydrogen atoms into account during the generation of intermediate chemical structures, the hydrogen atoms present in the substructures will be removed before the generation process begins. These substructures should be non-overlapping, i.e. they should not share any atoms. This limitation is due to the fact that our algorithm grows molecules by adding bonds and, if two atoms in different fragments were in fact the same atom, our algorithm would create bonds between those atoms, which would clearly lead to incorrect results.

In practice, multiple substructures can be available, but the user does not know if they overlap. This limitation can be circumvented by using the largest substructure as constraint for the generation and the remaining substructures as a posterior filtering, only keeping the molecules with those substructures.

By default, the structure generator returns the count of molecules it generated. Optionally, it can store all the molecules in an SDF file. If prescribed fragments are provided, OMG outputs only the molecules containing such fragments. We have opted to use SDF as our input and output format, but via CDK, other formats can easily be implemented in OMG.

### Data

As mentioned in the introduction, the identification of the chemical structure of metabolites is one of the current bottlenecks of metabolomics. In this sense, a structure generator can contribute to overcome this bottleneck, since it can provide candidate structures for an unknown metabolite. Therefore, metabolites appear to be a relevant family of compounds to test our structure generator. A list of metabolites was selected and their elemental composition was compiled to evaluate the performance of our structure generator on different inputs. The source of the compounds employed was the Human Metabolome Database (HMDB) [34], which contains almost 8,000 metabolites and is the most comprehensive database of human metabolites. A study of the human metabolite space and the properties of the metabolites that occupy it, has been previously reported [28]. The selection criteria were to include cyclic and acyclic compounds, of different molecular weights, and containing different chemical elements like C, O, N, P, and S. A first test set included metabolites with C, O, and H, chemical elements with one valence. A second test set included metabolites with C,O,H and also chemical elements with multiple valences, like N, P, and S. Furthermore, for some of these metabolites, several substructures were drawn and provided to the structure generator as additional input. These substructures are easily identified by an expert from direct inspection of MS^2^ or MS^n^ experimental data. The aim was to assess the importance of having fragment information to reduce the list of generated structures.

## Results and discussion

### Structure generation from elemental formula

The algorithm presented in this work, the Open Molecule Generator, was tested and compared with the commercial structure generator, MOLGEN. Both generators take resonance into account producing all the contributing structures. As a result, the two resonant forms of benzene will be considered as different molecules. Both OMG and MOLGEN are not limited to acyclic structures [35,36],thus the two structure generators tested can generate molecules with rings. Furthermore, both tools generate molecules containing common chemical elements present in metabolites, like C, O, N, H, P, and S, and are not limited to only 4 chemical elements [36]. Both structure generators generate molecules for a given elemental composition by exhaustively producing all non-redundant chemical structures.

The number of molecules produced after using the elemental compositions of a diverse selection of metabolites containing only C, O and H, is presented in Table 2. For all these metabolites, the same number of molecules is generated by both generators. While both generators produce complete results, MOLGEN does it in less time. The time between initialization and finalization was measured using time functions in JAVA for OMG and equivalent functions in python for MOLGEN. We can observe in Table 2 the time in seconds to generate all the candidate structures and the time to generate each molecule in milliseconds. If we look at time per molecule, MOLGEN is 4 times faster than OMG for small molecules like pyruvic acid. For larger molecules MOLGEN obtains a constant time per molecule between 0.008 and 0.009 milliseconds, while OMG ranges from 18 to 45 milliseconds depending on the elemental composition. Lightweight profiling of OMG was performed using VisualVM (version 1.3.4), in order to have an understanding of the limiting points in the performance of OMG. The most relevant finding was that the canonization process, which uses Nauty, took half of the total running time.

**Table 2:** Number of chemical structures generated by OMG and MOLGEN using as input only the elemental compositions of metabolites containing C,O and H elements

**Structure**	**Name HMDB ID elemental composition**	**MOLGEN**			**OMG**		
		**# Candidate structures**	**Time (s)**	**Time per molecule (ms)**	**# Candidate structures**	**Time (s)**	**Time per molecule (ms)**
	Pyruvic acid HMDB00243 C3H4O3	152	0.129	0.849	152	0.509	3.349
	Malic acid HMDB00156 C4H6O5	8,070	0.222	0.028	8,070	27.074	3.355
	D-Xylose HMDB00098 C5H10O5	18,092	0.332	0.018	18,092	125.783	6.952
	D-Fructose HMDB00660 C6H12O6	267,258	2.381	0.009	267,258	5,035.371	18.841
	Sedoheptulose HMDB03219 C7H14O7	4,106,823	38.945	0.009	4,106,823	186,248.085	45.351
	Pectin HMDB03402 C6H10O7	3,183,337	26.512	0.008	3,183,337	46,320.522	14.551
	Galactonic acid HMDB00565 C6H12O7	767,569	6.957	0.009	767,569	22,475.987	29.282
	Galactaric acid HMDB00639 C6H10O8	8,568,129	78.354	0.009	8,568,129	186,730.365	21.794
	Cholic acid HMDB00619 C24H40O5	* More than 2,147,483,646	* not available	* not available	* More than 2,147,483,646	* not available	* not available
	Phenyllactic acid HMDB00779 C9H10O3	48,496,265	404.052	0.008	** More than 48,496,265	** not available	** not available

*Results were not generated due to excessive computational time needed to generate all the candidate structures. However, we expect OMG to generate more molecules than MOLGEN, due to the larger amount of atom types produced by OMG.

**Results were not generated due to excessive computational time needed to generate all the candidate structures.

We observed that MOLGEN stops the generation of molecules after two billion molecules, as it can be observed for a large molecule like cholic acid (Table 2). Since both generators produce the same molecules for elemental composition with C, O and H, we can only assume that more than two billion molecules could be generated. In the case of phenyllactic acid, MOLGEN produces more than 48 million molecules in 404 seconds. Due to excessive computational time, no results for this elemental composition are reported for OMG, though the same number of molecules is expected (if executed for enough time) as is the case for all the other elemental compositions in this subset.

As stated in Methods, both generators treat atoms having multiple valences in different ways, this is the reason to use a second set of molecules containing also N, P and S. The default valences used by MOLGEN for N is 3, for P is 3, and for S is 2, unless stated otherwise. The results for these molecules are presented in Table 3. As expected, the number of candidate structures differs between both generators. For the elemental composition of glycine, MOLGEN produces 84 molecules only with N valence 3 and 162 molecules only with N valence 5. For the same elemental composition, OMG produces 97 molecules with valence 3 and 5 for N, which include the 84 of MOLGEN N valence 3 and 13 additional molecules with valence 5, containing N with the atom types depicted in Table 1 for OMG-CDK. The difference in the number of candidate structures is larger for elemental compositions containing many atoms with multiple valences, as is the case of creatinine. For this metabolite, MOLGEN generates 93,323 candidate structures with the default valence 3 for N. On the contrary, OMG produces 303,601 candidate structures, containing N valence 3 and 5.

**Table 3:** Number of chemical structures generated by OMG and MOLGEN using as input only the elemental compositions of metabolites containing C, O, H, N, P and S elements

**Structure**	**Name HMDB ID elemental composition**	**MOLGEN**			**OMG**		
		**# Candidate structures**	**Time (s)**	**Time per molecule (ms)**	**# Candidate structures**	**Time (s)**	**Time per molecule (ms)**
	Glycine HMDB00123 C2H5NO2	N_3 84	0.118	1.405	97	0.452	4.660
		N_5 162	0.120	0.741			
	Acetyl-HMDB00532 C4H7NO3	18,469	0.282	0.015	26,530	126.117	4.754
	Phenylalanine HMDB00159 C9H11NO2	277,810,163	2227.796	0.008	* More than 277,810,163	* not available	* not available
	Glutamic acid HMDB00148 C5H9NO4	440,821	2.945	0.007	685,392	12,348.456	18.017
	Phosphoenolpyruvic acid HMDB00263 C3H5O6P	P_3 51,323	0.562	0.011	83,977	761.378	9.067
		P_5 129,421	1.398	0.011			
	Creatinine HMDB00562 C4H7N3O	93,323	0.933	0.010	303,601	3,921.157	12.915
	Guanidinoacetic acid HMDB00128 C3H7N3O2	45,626	0.585	0.013	124,808	1,962.532	15.724
	Cytosine HMDB00630 C4H5N3O	108,769	1.149	0.011	491,299	3,952.098	8.044
	Uric acid HMDB00289 C5H4N4O3	464,899,034	3488.097	0.008	* More than 464,899,034	* not available	* not available
	Histamine HMDB00870 C5H9N3	46,125	0.631	0.014	134,278	3,566.544	26.561
	D-Cysteine HMDB03417 C3H7NO2S	3,838	0.156	0.041	15,978	131.004	8.199
	p-Cresol sulfate HMDB11635 C7H8O4S	S_6	5078.132	0.009	* More than 82,000,000	* not available	* not available
		592,625,133					

* Results were not generated due to excessive computational time needed to generate all the candidate structures. We expect OMG to generate more molecules than MOLGEN, due to the larger amount of atom types produced by OMG.

In the case of phosphoenolpyruvic acid, we require P valence 5 to be considered. On the one hand, running MOLGEN with the default valence for P yields 51,323 candidate structures but the correct molecule is missing. On the other hand, forcing the valence of P to be 5, returns 129,421 candidate structures, with the correct molecule also produced but also an excessive quantity of unrealistic molecules due to unrealistic atom types for P. Alternatively, OMG generates 83,977 candidate structures with P valence 3 and 5, including the desired molecule, where all of them are valid molecules as defined by the CDK atom dictionary.

We observe in Table 3 that the running time per generated molecule now ranges between 0.008 and 0.041 milliseconds, while OMG requires between 4.8 and 26.6 milliseconds. Such difference in execution speed between MOLGEN and OMG makes that for some large elemental compositions, only results are reported for MOLGEN. This is the case of phenylalanine, uric acid and p-cresol sulfate. However, for these metabolites, we assume that the number of candidate structures would have been higher with OMG than the one reported by MOLGEN using the default valences.

### Structure generation from elemental formula and prescribed substructures

Structure generation is a combinatorial problem where the number of output molecules grows exponentially with to the number of input atoms. When using one or more prescribed substructures as input to the generators in addition to elemental composition, less candidate structures are obtained (Table 4). Whereas MOLGEN can only accept one substructure, OMG can accept multiple substructures as input with the constraint that these do not overlap, i.e., they should not share any atom. Phenylalanine is a good example how the number of generated structures can be reduced by using more prescribed substructures, as will be discussed below in more detail.

**Table 4:** Number of chemical structures generated by OMG and MOLGEN using as input an elemental composition and one or more prescribed and non-overlapping fragments

**Structure**	**Name HMDB ID elemental composition**	**Prescribed substructure(s)**	**MOLGEN**			**OMG**		
			**# Candidate structures**	**Time (s)**	**Time per molecule (ms)**	**# Candidate structures**	**Time (s)**	**Time per molecule (ms)**
	Glycine		6	0.167	27.833	6	0.539	89.833
	HMDB00123							
	C2H5NO2							
	D-Cysteine		100	0.193	1.930	210	3.177	15.129
	HMDB03417							
	C3H7NO2S							
	Phenylalanine		76,247	52.774	0.692	107,155	19386.019	180.916
	HMDB00159							
	C9H11NO2							
			* not possible	* not possible	* not possible	595	271.809	456.822
			* not possible	* not possible	* not possible	289	172.655	597.422
			* not possible	* not possible	* not possible	26	25.147	967.192
	Cholic acid		** not possible	** not possible	* not possible	334	120.519	360.835
	HMDB00619							
	C24H40O5							
			* not possible	* not possible	* not possible	2,505	119.418	47.672
	Malic acid		1,436	0.229	0.159	1,436	4.688	3.265
	HMDB00156							
	C4H6O5							
	Uric acid		150,114	962.016	6.409	6,069,863	155828.437	25.672
	HMDB00289							
	C5H4N4O3							
	Phenyllactic acid		21,040	15.674	0.745	26,164	163.904	6.264
	HMDB00779							
	C9H10O3							
			* not possible	* not possible	* not possible	525	3.973	7.568
	p-Cresol sulfate		S_6 13,177	65.667	4.983	13,177	63.047	4.785
	HMDB11635							
	C7H8O4S							
			S_6 70,330	94.898	1.349	17,232	1204.357	69.891

*MOLGEN can only accept one prescribed substructure, while OMG accepts multiple substructures, provided that these do not overlap, this is, they do not share any atom.

**MOLGEN is not able to generate molecules using this large substructure as input. The reason could not be found.

Substructure information is of great relevance for metabolomics experiments involving MS^n^ data, where often the only information available of an unknown metabolite that needs to be identified is the elemental composition and in some cases substructures. Provided that no database entries exist for this experimental information, one is forced to generate the structures via CASE. The inclusion of substructure information brings the list of candidate structures to a manageable size. For p-cresol sulfate, using the sulfate group with both generators as prescribed substructure, produces 13,177 molecules. When benzene is the prescribed substructure, OMG generates 17,232 candidate structures and MOLGEN 70,330, all containing sulfur with valence 6, hence the difference between both generators.

Whereas only the elemental composition of phenylalanine as input generates 277 million structures with MOLGEN and for OMG an even higher number of candidate structures is expected as both nitrogen valences of 3 and 5 are taken into account (Table 3), using benzene as a substructure provides only 107,155 (OMG) and 76,247 (MOLGEN) candidate structures (Table 4). The number of generated molecules for the elemental composition of phenylalanine is even further reduced by prescribing multiple fragments as input: OMG outputs 595 molecules when provided with two fragments and 289 molecules for three fragments (Table 4). The use of large fragments yields the larger reduction in output molecules, as it can be seen for the last example of phenylalanine, where two big fragments describe most of its structure and return only 26 chemical structures.

For larger molecules containing ten or more carbon atoms, which is a common situation in chemistry, it is not practical for the identification of metabolites to exhaustively generate candidate structures without using substructure constraints, with MOLGEN and OMG, due to the large number of results. Using the elemental composition of a large metabolite like cholic acid, both structure generators cannot produce all possible candidate structures, which are expected in the order of billions. This was only possible using substructure information to reduce the size of the search tree: when providing a substructure that describes a large part of the molecule, OMG generates only 334 structures (Table 4). When using two substructures, OMG returned 2,505 candidate structures. However, MOLGEN was unable to return results using the same large substructure or two substructures as an input and the reason could not be found by us.

The use of prescribed substructures affected the running time of both generators. For MOLGEN, the time per molecule ranged between 0.16 and 27.8 milliseconds, which represents in some cases a 10,000-fold increase in computation time compared to using only elemental compositions. Concerning OMG, the time per molecule ranged between 3.3 and 967. 2 milliseconds, a 100-fold increase in running time. Despite this deterioration of execution time, the advantage of using one or ideally multiple prescribed substructures is clear: the number of candidate substructures is significantly reduced and the total time to calculate candidate structures is also reduced compared to not using any substructure.

The results here presented show that if we want MOLGEN to generate the correct molecule when the valence of some atoms is not the default one, like phosphoenolpyruvic acid or p-cresol sulfate, we need to know the valence in advance. Otherwise, MOLGEN should be executed using all possible valences for all atoms. This limitation is not present in OMG, which can produce different valences in the same execution. Unfortunately, the atom dictionary provided by CDK is not comprehensive concerning non-standard valences. On the positive side, the dynamic open source community of CDK keeps adding new atom types with each release of the library and we expect that this will improve the capabilities of OMG. This open source nature of CDK allows users to suggest or implement new atom types.

The generation of the molecules in the Open Molecule Generator has the shape of a tree. As stated by McKay [23], the check for canonical augmentation is branchindependent, which would allow to process branches of the generation trees in parallel. Theoretically the algorithm allows for parallelization, in practice this has not been implemented but it is one future extension of this work.

However, we have observed that OMG is in most of the cases slower than MOLGEN and this fact was more noticeable when generating millions of candidate molecules. The speed of OMG could be improved and we see several possibilities to achieve this, i.e. the use of a different canonizer or a less computationally demanding canonicity test for intermediate chemical structures, could significantly speed up the execution. Actually, obtaining millions of molecules as a result, quickly or slowly, is not desirable, but ideally, the goal of metabolite identification is to obtain a list of candidate structures that is short in order to examine it and find the structure belonging to the unknown metabolite. Exhaustive profiling, covering both on execution time and memory use, would be beneficial to discover improvement points for OMG. Fortunately, OMG allows multiple prescribed substructures and can handle large fragments, which reduced the number of generated molecules significantly. Handling multiple substructures allows OMG to provide a short list of candidate structures and additionally, its open source nature permits users to implement specific constraints to further reduce the candidate list, both during and after the generation process. Examples of such constraints would reject intermediate chemical structures with high steric energy values or other physicochemical properties. Therefore we expect OMG to be useful in different application areas and its functionality to be extended in the near future.

## Conclusion

In this work we have presented the Open Molecule Generator, to the best of our knowledge, the first implementation to chemical structure generation of the Canonical Path Augmentation approach, originally designed for simple graph enumeration adding vertices. We have adapted it to generate organic chemical structures and extended so that (i) it grows molecules by adding bonds, (ii) it can handle multigraphs, and (iii) accepts one or multiple non overlapping prescribed substructures. In addition, this is the first open source implementation of a deterministic structure generator. This will enable future developments like parallelization or the inclusion of constraints that are specific to the class of compounds being generated.

Our results show that the implementation of our algorithm generates all possible and valid chemical structures for a given elemental composition and optionally prescribed substructures. It is as complete as the best commercially available generator. Moreover, the current implementation of the OMG program presents an extra advantage over existing generators when large or multiple fragments are available to be used as constraints: we have demonstrated the benefit of incorporating constraints to reduce the number of output molecules significantly. The ability of OMG to generate multiple valences for an atom has proven to be useful as often no prior information is known on the desired chemical elements and multiple valences of an element can be present in a molecule. When compared to MOLGEN, the only disadvantage of OMG is its speed, which is more severe when using only elemental compositions and less when including prescribed substructures. This issue will be addressed in future improvements of the program. We expect this tool to be used in various fields, one of them being metabolomics, where there is a clear need for flexible structure generators. We have successfully used OMG to propose candidate structures using prescribed substructures, in several on-going metabolite identification projects in our lab.

## Availability and requirements

**Project name:** openmg

**Project home page:**http://sourceforge.net/p/openmg

**Operating system:** Linux 64bits, Linux 32bits, Mac OS X

**Programming languages:** Java, C

Other requirements (if compiling):

**License:** GNU AGPL v3

**Any restrictions to use by non-academics**: None other than those specified by the license

## Algorithms

1: ***generate*(M)**

2:  **If ***saturated*(M) AND *are_all_H_used*(M)

3:    **If ***connected_fragments*(M) == 1

4:     *store_to_file*(M)

5:     Nmols = Nmols + 1

6:     **If** degree(M) < max_degree(M)

7:       *generate*(M)

8:     **Endif**

9:    **Endif**

10:  **Else**

11:   New Map

12:   List_of_bonds = *extend*(M)

13:   **Foreach** bond **in** list_of_bonds

14:     M’ = *add_bond*(bond,M)

15:     canonM’ = *canonize*(M’)

16:     **If** not *is_present*(map,canonM’)

17:       *add*(map,canonM’)

18:       **If ***is_canonical_augmentation* (canonM’,M’,M)

19:          *generate*(M’)

20:       **EndIf**

21:     **EndIf**

22:   **End**

23:  **EndIf**

24: **End**

Algorithm 1

1: **Is_canonical_augmentation(canonM’, M’, M)**

2:  last_bond = *get_last_bond*(canonM’)

3:  M” = *remove_bond*(M’, last_bond)

4:  **return ***are_the_same*(M”, M)

5: **End**

Algorithm 2

1: **Is_canonical_augmentation_fragments(canonM’, M’, M)**

2:  last_bond = *get_last_bond*(canonM’)

3:  **While ***bond_belongs_to_fragment*(last_bond, canonM’)

4:     last_bond = *get_previous_bond*(canonM’)

5:  **Endwhile**

6:  M” = *remove_bond*(M’, last_bond)

7:  **return ***are_the_same*(M”, M)

8: **End**

Algorithm 3

## Competing interests

The authors declare that they have no competing interests.

## Authors’ contributions

JEP designed and implemented the software, and drafted most of the manuscript. MRC and DF contributed to the implementation of the software. JLF contributed to the design of the algorithm and supervised specific parts of the project. TR, LC and TH supervised specific parts of the project, fed it with new ideas, and participated in testing the software. All authors approved the final manuscript.
